# Angiotensin Type 2 and Mas Receptor Activation Prevents Myocardial Fibrosis and Hypertrophy through the Reduction of Inflammatory Cell Infiltration and Local Sympathetic Activity in Angiotensin II-Dependent Hypertension

**DOI:** 10.3390/ijms222413678

**Published:** 2021-12-20

**Authors:** Giovanna Castoldi, Raffaella Carletti, Silvia Ippolito, Andrea Stella, Gianpaolo Zerbini, Sara Pelucchi, Giovanni Zatti, Cira R. T. di Gioia

**Affiliations:** 1 Dipartimento di Medicina e Chirurgia, Università degli Studi di Milano-Bicocca, 20900 Monza, Italy; andrea.stella@unimib.it (A.S.); sara.pelucchi@unimib.it (S.P.); giovanni.zatti@unimib.it (G.Z.); 2 Dipartimento di Medicina Traslazionale e di Precisione, Sapienza Universita’ di Roma, 00161 Rome, Italy; raffaella.carletti@uniroma1.it; 3 Laboratorio Analisi Chimico Cliniche, Ospedale San Gerardo, ASST Monza, 20900 Monza, Italy; s.ippolito@asst-monza.it; 4 Unita Complicanze del Diabete, IRCCS Istituto Scientifico San Raffaele, 20132 Milan, Italy; zerbini.gianpaolo@hsr.it; 5 Clinica Ortopedica, Ospedale San Gerardo, ASST Monza, 20900 Monza, Italy; 6 Dipartimento di Scienze Radiologiche, Oncologiche e Anatomopatologiche, Istituto di Anatomia Patologica, Sapienza Universita’ di Roma, 00161 Rome, Italy; cira.digioia@uniroma1.it

**Keywords:** AT2 receptor agonist, angiotensin II, angiotensin 1-7, hypertension, myocardial hypertrophy, myocardial fibrosis, rats

## Abstract

Compound 21 (C21), an AT2 receptor agonist, and Angiotensin 1-7 (Ang 1-7), through Mas receptor, play an important role in the modulation of the protective arm of the renin-angiotensin system. The aim of this study was to investigate in an experimental model of angiotensin II-dependent hypertension whether the activation of the potentially protective arm of the renin-angiotensin system, through AT2 or Mas receptor stimulation, counteracts the onset of myocardial fibrosis and hypertrophy, and whether these effects are mediated by inflammatory mechanism and/or sympathetic activation. Sprague Dawley rats (*n* = 67) were treated for 1 (*n* = 25) and 4 (*n* = 42) weeks and divided in the following groups: (a) Angiotensin II (Ang II, 200 ng/kg/min, osmotic minipumps, sub cutis); (b) Ang II+Compound 21 (C21, 0.3 mg/kg/day, intraperitoneal); (c) Ang II+Ang 1-7 (576 µg/kg/day, intraperitoneal); (d) Ang II+Losartan (50 mg/kg/day, per os); (e) control group (physiological saline, sub cutis). Systolic blood pressure was measured by tail cuff method and, at the end of the experimental period, the rats were euthanized and the heart was excised to evaluate myocardial fibrosis, hypertrophy, inflammatory cell infiltration and tyrosine hydroxylase expression, used as marker of sympathetic activity. Ang II caused a significant increase of blood pressure, myocardial interstitial and perivascular fibrosis and myocardial hypertrophy, as compared to control groups. C21 or Ang 1-7 administration did not modify the increase in blood pressure in Ang II treated rats, but both prevented the development of myocardial fibrosis and hypertrophy. Treatment with losartan blocked the onset of hypertension and myocardial fibrosis and hypertrophy in Ang II treated rats. Activation of AT2 receptors or Mas receptors prevents the onset of myocardial fibrosis and hypertrophy in Ang II-dependent hypertension through the reduction of myocardial inflammatory cell infiltration and tyrosine hydroxylase expression. Unlike what happens in case of treatment with losartan, the antifibrotic and antihypertrophic effects that follow the activation of the AT2 or Mas receptors are independent on the modulation of blood pressure.

## 1. Introduction

The renin angiotensin system plays a key role in blood pressure control, electrolyte balance and cardiovascular homeostasis. Angiotensin II (Ang II) has historically been considered the main effector of the renin-angiotensin system and nowadays drugs interfering with the renin angiotensin system, as ACE-inhibitors or AT1 blockers, represent the therapeutic cornerstone to treat hypertension and to counteract cardiovascular and renal diseases [1,2]. Increased blood pressure values are the key factors in the development of myocardial hypertrophy and fibrosis, which represent the main cardiac damages induced by hypertension [3,4].

Despite the wide use of antihypertensive therapy, it is not always possible counteract the development of organ damage, which is determined by systemic, mainly hemodynamic, and tissue factors.

Ang II, in addition to its classical and well-known hemodynamic action, has pro-inflammatory effects, increases sympathetic activity, and has pro-fibrotic/hypertrophic cellular effects [5,6,7], mainly mediated by AT1 receptors. In addition to the classical pathway of the RAS system (Renin/ACE/Angiotensin II/AT1 receptor), the existence of a parallel ACE2/Ang 1-7/Mas/AT2 receptors pathway has been demonstrated in the last decades [8,9]. It is generally assumed that ACE2/Ang 1-7/Mas/AT2 receptors pathway may counteract the effects of the classical RAS pathway, promoting anti-inflammatory, anti-hypertrophic and anti-fibrotic effects, and it is therefore defined as the “protective arm” of RAS [8,9,10,11,12]. In fact, activation of AT2 receptors improves cardiac function in rat with myocardial infarction [13,14], prevents the development of myocardial hypertrophy in type 2 diabetic rats [15], vascular stiffness in an experimental model of N-nitro-l-arginine-methyl ester-induced hypertension [16], myocardial fibrosis in stroke-prone spontaneously hypertensive rats [17], salt induced myocardial hypertrophy in Wistar rats [18], and reduces myocardial fibrosis in experimental model of cyclosporine nephropathy [19]. Similarly, Ang 1-7 administration prevents cardiac remodeling in Ang II treated rats without modifying AT1 and AT2 receptor population [20], and attenuates myocardial hypertrophy by a mitochondrial ROS-dependent mechanism [21]. In exercise-trained two kidney-1 clip hypertensive rats Ang 1-7 administration reduces hypertension, myocardial fibrosis and hypertrophy [22], while in diabetic mice ameliorates diabetic cardiomyopathy and diastolic dysfunction by reducing lipotoxicity and inflammation [23] and prevents cardiac fibrosis in Doca-salt hypertension [24].

In the present study we investigated potential mechanisms involved in the beneficial effects of AT2 or Mas receptor agonists on myocardial fibrosis and hypertrophy in Ang II- dependent hypertension. Here we show that cardioprotective effects of AT2 and Mas receptor stimulation are mediated by local anti-inflammatory mechanisms and by a reduction of sympathetic activity, and are independent on the modulation of the increase of blood pressure caused by Ang II administration.

## 2. Results

### 2.1. Effects of C21 and Ang 1-7 Administration on Systolic Blood Pressure, Serological Parameters, Glomerular Filtration Rate, Heart Weight and Heart/Body Weight Ratio in Ang II Treated Rats for 1 and 4 Weeks

Angiotensin II administration for 1 and 4 weeks caused a significant increase in blood pressure in Ang II treated rats as compared to control rats (Table 1 and Table 2). C21 or Ang 1-7 treatment did not significantly modify the increase of blood pressure caused by Ang II administration (Table 1 and Table 2).

Losartan administration blocked the Ang II mediated increase in blood pressure after 1 week of treatment (Table 1) and this effect was maintained for the subsequent 4 weeks (Table 2). Non-fasting plasma glucose, sodium, potassium, calcium, phosphate, total cholesterol, triglycerides, C reactive protein and monocyte chemoattractant protein-1 were not significantly modified by the different treatments both in the short and long term protocol (Table 1 and Table 2). As compared to control group, Ang II administration for 4 weeks did not change the CRP values in the group treated with Ang II alone, while there was a trend towards an increase in the groups treated with Ang II+C21, Ang II+Ang 1-7 and Ang II+losartan (Table 2).

Ang II administration for 1 week did not modify body weight in Ang II treated rats, treated or not with C21, or Ang 1-7, or losartan as compared to control rats (Table 1). Body weight was lower in Ang II, Ang II+C21, Ang II+Ang 1-7, Ang II+Losartan treated rats for 4 weeks as compared to control rats (Table 2). Ang II administration for 1 week caused a slight increase in heart/body weight ratio in Ang II, Ang II+C21 and Ang II+Ang 1-7 treated rats as compare to control rats (Table 1). Ang II administration for 4 weeks caused a significant increase of heart/body weight ratio in Ang II, Ang+C21 and Ang II+Ang 1-7 treated rats as respect to control animals (Table 2). Losartan treatment prevented the increase of heart/body weight ratio both after 1 and 4 weeks of administration in Ang II treated rats (Table 1 and Table 2). Ang II administration for 1 week, alone or in combination with C21, Ang 1-7 or losartan, did not modify glomerular filtration rate as compared to control rats (Table 1), while, after 4 weeks, Ang II caused a slight decrease in glomerular filtration rate, regardless the treatments (Table 2).

### 2.2. Effects of C21 and Ang 1-7 Administration on Myocardial Fibrosis and Hypertrophy in Ang II Treated Rats for 1 and 4 Weeks

**Figure 1:**
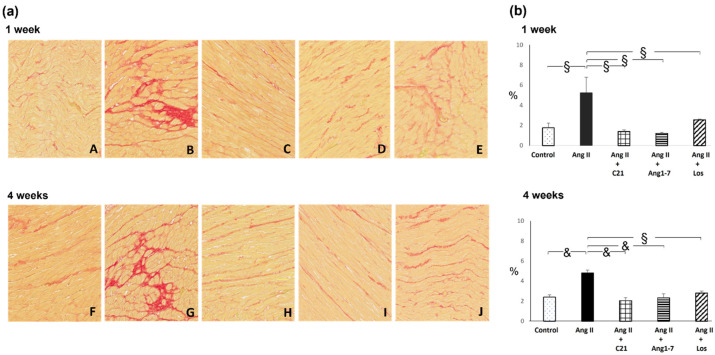
Myocardial interstitial fibrosis. Effects of C21 and Ang 1-7 administration on myocardial interstitial fibrosis in Ang II treated rats for 1 and 4 weeks of treatment. (**a**). Representative photomicrographs of interstitial fibrosis in control (A,F), Ang II (B,G), Ang II + C21 (C,H), Ang II + Ang 1-7 (D,I) and Ang II + Los (E,J) treated rats (×20 magnification) after 1 and 4 weeks of treatment. (**b**). Quantification of interstitial fibrosis in the different groups of rats. Data are means ± SEM. &: *p* <  0.01. §: *p*  <  0.0001.

**Figure 2:**
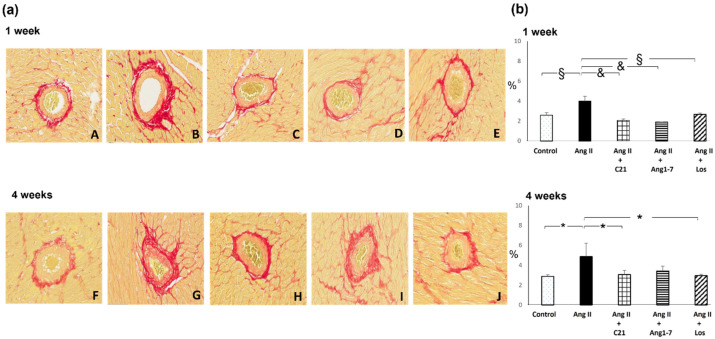
Myocardial perivascular fibrosis. Effects of C21 and Ang 1-7 administration on myocardial perivascular fibrosis in Ang II treated rats for 1 and 4 weeks. (**a**). Representative photomicrographs of perivascular fibrosis in control (A,F), Ang II (B,G), Ang II + C21 (C,H), Ang II + Ang 1-7 (D,I) and Ang II + Los (E,J) treated rats (×20 magnification), after 1 and 4 weeks of treatment. (**b**). Quantification of perivascular fibrosis in the different groups of rats. Data are means ± SEM. *: *p* <  0.05. §: *p*  <  0.01. &: *p*< 0.0001.

Ang II administration for 1 and 4 weeks caused a significant increase in myocardial fibrosis, both at interstitial and perivascular level (Figure 1 and Figure 2).

Treatment with C21, or Ang 1-7 or losartan blocked the increase of myocardial interstitial and perivascular fibrosis caused by Ang II administration.

The antifibrotic effect of the different treatments was evident just after 1 week, and was mantained during the 4 weeks of Ang II administration (Figure 1 and Figure 2). The antifibrotic effect of C21 or Ang 1-7 was similar to that obtained by blocking the AT1 receptors with losartan, which also prevented the increase of blood pressure in Ang II treated rats at both after 1 and 4 weeks (Table 1 and Table 2).

**Figure 3:**
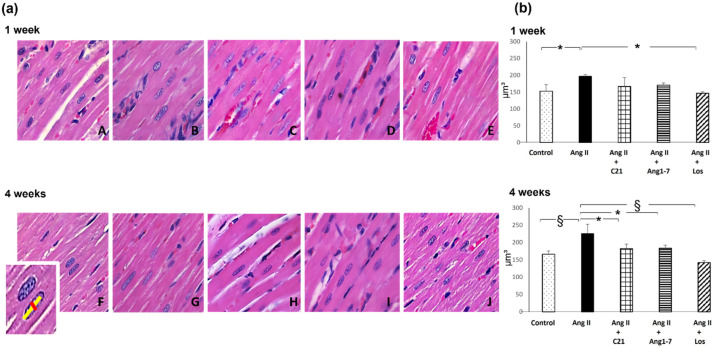
Myocardial hypertrophy. Effect of C21 and Ang 1-7 administration on myocardial hypertrophy in Ang II treated rats for 1 and 4 weeks. (**a**). Representative images of histological left ventricular sections in control (A,F), Ang II (B,G), Ang II + C21 (C,H), Ang II + Ang 1-7 (D,I) and Ang II + Losartan (E,J) treated rats (H&E, 40X magnification), respectively at 1 and 4 weeks of treatment, that show hypertrophy with related enlarged volume of cardiomyocyte nuclei in Ang II treated rats (B,G) as compared to control rats (A,F) at 1 and 4 weeks of treatment. C21 (H), Ang 1-7 (I) and losartan (J) administration for 4 weeks in Ang II treated rats prevented the development of myocardial hypertrophy. (**b**). Quantification of myocardial hypertrophy in the different groups of rats. Data are means ± SEM. *: *p* <  0.05. §: *p* <  0.01.

Myocardial hypertrophy was significantly increased in Ang II treated rats as compared to control rats and it was prevented by C21, or Ang 1-7 or losartan administration (Figure 3). After 4 weeks of treatment, myocardial nuclear cardiomyocyte volume was significantly higher in Ang II treated rats as compared to control, Ang II+C21, Ang II+Ang 1-7 and Ang II+Los treated rats (Figure 3).

### 2.3. Effects of C21 and Ang 1-7 Administration on Myocardial Inflammatory Cell Infiltration and Tyrosine Hydroxylase Expression in Ang II Treated Rats for 1 and 4 Weeks

**Figure 4:**
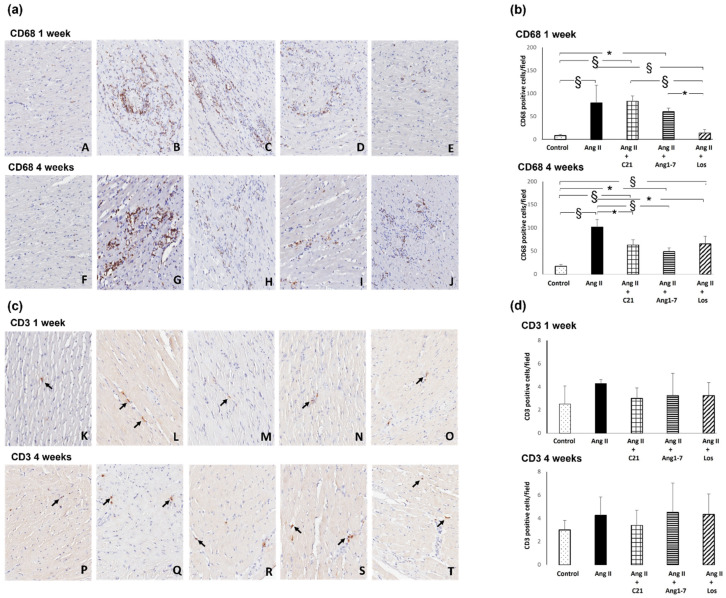
Myocardial inflammatory cell infiltration. Effects of C21 and Ang 1-7 administration on myocardial inflammatory cell infiltration in Ang II treated rats for 1 and 4 weeks. (**a**). Immunohistochemical identification of interstitial monocyte/macrophage infiltration (brown reaction) in control (A,F), Ang II (B,G), Ang II + C21 (C,H), Ang II + Ang 1-7 (D,I) and Ang II + Los (E,J) treated rats (×20 magnification), at 1 and 4 weeks of treatment. (**b**). Quantification of staining of myocardial monocyte/macrophage inflammatory cells in the different groups of rats. Data are means ± SEM. *: *p*  <  0.05. §: *p*  <  0.01. (**c**). Immunohistochemical identification of interstitial T-lymphocyte infiltration (brown reaction) in control (K,*P*), Ang II (L,Q), Ang II + C21 (M,R), Ang II + Ang 1-7 (N,S) and Ang II + Los (O,T) treated rats (×20 magnification), at 1 and 4 weeks of treatment. (**d**). Quantification of staining of myocardial T-lymphocyte inflammatory cells in the different groups of rats. Data are means ± SEM.

Ang II administration for 1 week caused a significant increase in myocardial monocyte/macrophage (CD68 positive cells) infiltration in Ang II treated rats (Figure 4a,b). C21 or Ang 1-7 administration did not modify the dysfunction that was instead prevented by losartan treatment (Figure 4a,b).

Ang II administration for 4 weeks caused a significant increase in myocardial monocyte/macrophage infiltration in Ang II treated rats (Figure 4a,b). C21, or Ang 1-7 or losartan administration blunted the Ang II dependent increase of myocardial inflammatory cell infiltration as compared to Ang II treated rats. After 4 weeks of Ang II administration monocyte/macrophage infiltration in myocardial tissue in Ang II+C21, Ang II+Ang 1-7 and Ang II+losartan treated rats was higher as respect to control rats (Figure 4a,b).

Altogether, as compared to control rats, Ang II administration or the combined treatments for 1 or 4 weeks did not significant modify the presence of T-lymphocytes (CD3 positive cells), which were sporadically present in the myocardial tissue (Figure 4c,d).

**Figure 5:**
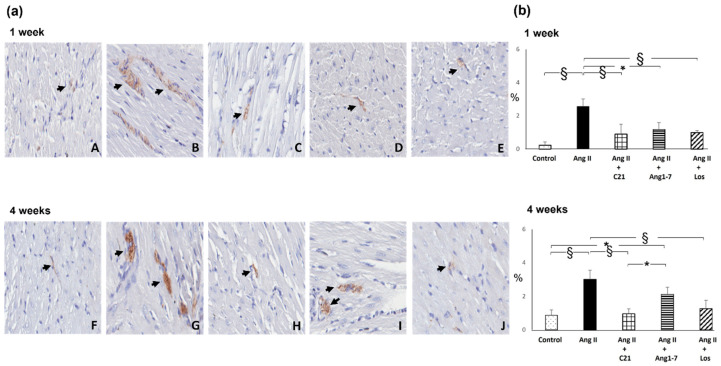
Myocardial tyrosine hydroxylase expression. Effects of C21 and Ang 1-7 administration on myocardial tyrosine hydroxylase expression in Ang II treated rats for 1 and 4 weeks. (**a**). Immunohistochemical identification of tyrosine hydroxylase expression at intraparenchymal nerve fiber level (brown reaction) in control (A,F), Ang II (B,G), Ang II + C21 (C,H), Ang II + Ang 1-7 (D,I) and Ang II + Los (E,J) treated rats (×20 magnification) at 1 and 4 weeks of treatment. (**b**). Quantification of staining of myocardial tyrosine hydroxylase expression in the different groups of rats. Data are means ± SEM. *: *p* <  0.05. §: *p* <  0.01.

Ang II administration for 1 week caused a significant increase in myocardial tyrosine hydroxylase protein expression (Figure 5). C21, Ang 1-7 or losartan treatment prevented this increase (Figure 5).

As compared to control rats, Ang II administration for 4 weeks caused a significant increase in myocardial tyrosine hydroxylase expression (Figure 5), which was completely blocked by C21 or losartan treatment, but not by Ang 1-7 administration (Figure 5).

**Figure 6:**
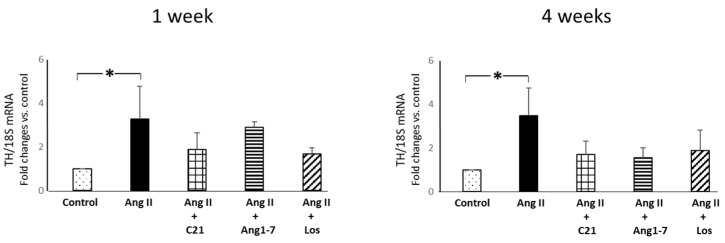
Myocardial mRNA tyrosine hydroxylase expression. Effects of C21 and Ang 1-7 administration on myocardial mRNA tyrosine hydroxylase expression in Ang II treated rats for 1 and 4 weeks. Data are mean ± SEM. *: *p*< 0.05.

Ang II administration for 1 and 4 weeks caused a significant increase in myocardial tyrosine hydroxylase (TH) mRNA expression (Figure 6) in Ang II treated rats, but not in Ang II+C21, Ang II+Ang 1-7 and Ang II +losartan treated rats, as compared to control rats (Figure 6).

## 3. Discussion

The results of this study demonstrate that the activation of AT2 receptor by C21 or the activation of Mas receptor by Ang 1-7 prevented the onset of myocardial fibrosis and hypertrophy in Ang II treated rats. These effects, which occur in presence of Ang II-induced hypertension, are mediated by the reduction of inflammatory cells infiltration in the myocardium and by the reduction of tyrosine hydroxylase expression, an established marker of the sympathetic activity. Different from the blockade of AT1 receptor by losartan, activation of AT2 or Mas receptors by C21 and Ang 1-7 treatment, respectively, did not reduce the marked increase of blood pressure caused by Ang II administration (Table 1 and Table 2), but were nevertheless as effective as losartan in the prevention of myocardial fibrosis and hypertrophy. In our experimental conditions, while in the short-term protocol (1 week of treatment) no changes in glomerular filtration rate occur (Table 1), Ang II administration for 4 weeks, alone or in combination with AT2 or Mas receptor agonists or with AT1 receptor antagonist, caused a slight but significant decrease in glomerular filtration rate (Table 2). Although the investigation of renal function goes beyond the aim of the present study, this finding should be considered in the use of these treatments in conditions of Ang II-dependent hypertension.

Hypertension is an important factor for the development of myocardial hypertrophy and fibrosis. In our experimental conditions however, AT2 receptor activation (by intraperitoneal injection of C21) promoted cardioprotection even in absence of the normalization of blood pressure. These results are in line with previous studies in different experimental models of hypertension, such as stroke-prone spontaneously hypertensive rats [17,25], spontaneously hypertensive rats [26], renal hypertension [27], L-NAME induced hypertension [16] in which peripheral administration of AT2 receptor agonist did not significantly modify blood pressure, but promote organ protection at vascular [16], renal [17,25,26,27], and myocardial level [17,25]. Similarly, in our experimental conditions, Ang 1-7 administration did not normalize blood pressure, but was effective to promote cardioprotection, confirming previous results [20,24].

Altogether the present results are in line with the hypothesis that the antifibrotic and antihypertrophic effects that result from the activation of the AT2 receptors or the Mas receptors involve multiple mechanisms different from the hemodynamic mechanism [11,28,29,30].

Ang II administration causes an increase in inflammatory infiltrates and sympathetic activity in the myocardium, which, along with the increased blood pressure, play a key role in the development of fibrosis and hypertrophy.

In particular, the monocyte/macrophage infiltrate represents the key factor for the development of fibrosis [31], while the activation of sympathetic activity at a local level contributes to the development of myocardial hypertrophy [32].

In our experimental conditions, the administration of Ang II for 1 week increased the inflammatory infiltrate, characterized by monocyte/macrophage cells, in the myocardium even in the presence of the co-administration of C21 and Ang 1-7. On the contrary, losartan blocked inflammatory infiltrates in the myocardium after 1 week of co-administration with Ang II. Interestingly, after 4 weeks of Ang II administration the monocyte/macrophage infiltration was reduced in the groups treated with C21, Ang 1-7, and losartan as compared to the group treated with Ang II alone, but these same parameters were nonetheless increased as compared to the control group. Finally, in rats that received Ang II for 4 weeks, the simultaneous treatment with Ang 1-7 did not normalize the myocardial protein expression of tyrosine hydroxylase after 4 weeks of co-administration Ang II+Ang 1-7, while C21 and losartan maintained their full preventive effect by blocking tyrosine hydroxylase protein expression over time.

Of interest, our data suggest that the activation of the AT2 or Mas receptors counteracts the development of myocardial fibrosis and hypertrophy through a combined action in reducing inflammation and sympathetic activity in the heart. In our experimental protocols, in presence of up to 4 weeks of administration of Ang II, the activation of either AT2 or Mas receptors was as effective in preventing fibrosis and myocardial hypertrophy as in case of blockade of AT1 receptors. However, we cannot exclude that if the Ang II profibrotic and prohypertrophic stimulus is prolonged for longer time, the single treatment with AT2 or Mas receptor agonist could be sufficient to contrast the onset of myocardial damage. Thus, combined administration of these two RAS protective agonists could be necessary in association with AT1 receptor blockade to better control myocardial fibrosis and hypertrophy, as also demonstrated in different organs of different experimental models [16,17,33].

In conclusion, these results support the hypothesis that the activation of the potentially protective arm of the renin-angiotensin-system could be a new therapeutic strategy to counteract myocardial organ damage in arterial hypertension.

## 4. Materials and Methods

### 4.1. Experimental Model of Ang II-Dependent Hypertension

Experiments were performed in male Sprague–Dawley rats (body weight 150–175 g) in accordance with Guide for the Care and Use of Laboratory Animals published by the US National Institutes of Health (NIH Publication No. 85-23, revised 1996). Animal husbandry was in conformity with the Institutional Guidelines in compliance with National laws and policies (D.L.n. 116, Gazzetta Ufficiale della Repubblica Italiana, suppl.40, 18 February 1992). Animals were individually housed in cages (or in metabolic cages at the end the experimental protocols to perform 24 h urine collection) in a temperature controlled room with a 12:12 light:dark cycle, with free access to a standard rat chow and tap water. Body weight (BW, g) was measured once a week. Before the start of the experimental period, rats were accustomed to measuring blood pressure with the tail cuff method (BP Recorder, Ugo Basile Instruments, Gemonio, VA, Italy). Systolic blood pressure was measured in the non-anaesthetized rat, placed in a restrainer and heated at 28 °C for 20–30 min to promote vasodilation. An expert investigator performed blood pressure measurements, at the beginning of any treatment and at the end of the experimental protocols. Each blood pressure value represents the average of 6 recordings [34].

Ang II (or saline in control groups) administration for 1 week (short-term protocol) and 4 weeks (long-term protocol) was administered through osmotic minipumps (Alzet 2001 and 2004, Cupertino, CA, USA), subcutaneously implanted in infrascapular side under sodium pentobarbital anesthesia (40 mg/kg/i.p.) [34].

For short-term protocol (1 week) Sprague-Dawley rats (*n* = 25) were divided in the following groups: control (saline treated group, *n* = 5); Ang II (200 ng/kg/min, *n* = 5; Sigma, Darmstadt, Germany); Ang II plus AT2 receptor agonist (Compound 21, C21, 0.3 mg/kg/day, intraperitoneal injection, *n* = 5; Vicore Pharma, Göteborg, Sweden); Ang II plus Angiotensin 1-7 (Ang 1-7, 576 µg/kg/day, intraperitoneal injection, *n* = 5; Bachem, Bubendorf, Switzerland) [35,36]; Ang II plus losartan (Los, 50 mg/kg/day, in drinking water, *n* = 5).

For long-term protocol (4 weeks) Sprague-Dawley rats (*n* = 42) were divided in the following groups: control (saline treated group, *n* = 8); Ang II (200 ng/kg/min, *n* = 6); Ang II plus AT2 receptor agonist (Compound 21, C21, 0.3 mg/kg/day, intraperitoneal injection, *n* = 10); Ang II plus Angiotensin 1-7 (Ang 1-7, 576 μg/kg/day, intraperitoneal injection, *n* = 10); Ang II plus losartan (Los, 50 mg/kg/day, in drinking water, *n* = 8).

Non-fasting plasma glucose (mg/dL), sodium (mEq/L), potassium (mEq/L), calcium (mg/dL), phosphate (mg/dL), total cholesterol (mg/dL), triglycerides (mg/dL), were measured by colorimetric technique on Cobas Roche (Mannheim, Germany). Glomerular filtration rate (GFR, mL/min) was evaluated as creatinine clearance. Plasma and urinary (24 h urine collection) creatinine was measured by colorimetric technique on Cobas Roche (Mannheim, Germany). Plasma C reactive protein (CRP, µg/mL; Rat C Reactive Protein, Merck-Sigma, Darmstadt, Germany) and monocyte chemoattractant protein-1 (MCP-1, pg/mL; Rat Monocyte Chemoattractant Protein -1, Merck-Sigma, Darmstadt, Germany) were measured by Elisa arrays, following manufacturer’s instructions.

At the end of the experimental periods, rats were euthanized by an overdose of anesthesia. The hearts were immediately excised, weighted and sectioned. The apex of the heart was cut, immediately frozen in liquid nitrogen and stored at −80 °C until total RNA extraction. The heart, sectioned into three transverse slices from the apex to the base, was fixed with 10% formalin, embedded in paraffin and used for light microscopic examination and morphometric analysis of myocardial fibrosis and hypertrophy. Myocardial inflammatory cell infiltration and tyrosine hydroxylase expression were evaluated by immunohistochemistry. Myocardial tyrosine hydroxylase mRNA expression was evaluated by realtime PCR.

### 4.2. Morphometric Analysis of Myocardial Interstitial and Perivascular Fibrosis

For all rats the coronal cardiac sections (3 µm) were deparaffinized, rehydrated, and stained both with hematoxylin–eosin (H&E) and Sirius Red, a collagen-specific stain. Changes in cardiac morphology were assessed by light microscopic analysis of H&E-stained sections for each group studied. The Sirius Red-stained sections were used for morphometric analysis of myocardial fibrosis, as previously described [19]. Briefly, all slides with Sirius Red-stained myocardial sections were captured with Aperio scanner (Leica Biosystems, Buccinasco, MI, Italy) and then 20 randomly selected images without vessels (×20 magnification) were analyzed to evaluate interstitial collagen volume fraction with a computerized imaging software (Image J-win32, NIH, Bethesda, MD, USA) and expressed as the ratio between red-stained interstitial area and the total area of the heart section. For analysis of perivascular fibrosis, 10 vessels were randomly selected at ×40 magnification from cardiac section of each rat. The image analysis of intraparenchymal vessels was performed in semiautomatic fashion. Only the collagen immediately surrounding each intraparenchymal vessel was considered to represent perivascular collagen deposition. The perivascular collagen volume fraction was expressed as the ratio of collagen area surrounding the traced vessel to total cross-sectional area, to correct differences in vessel size. Image analysis was performed by two pathologists blinded to the experimental source of the samples [19].

### 4.3. Histological Analysis and Morphometric Evaluation of Myocardial Hypertrophy

Histological cardiac sections (3 µm) stained with hematoxylin-eosin were used to evaluate cardiomyocyte hypertrophy. All slides were captured with Aperio scanner (Leica Biosystems) and then 5 images (×20 magnification) were randomly selected and analyzed to evaluate myocardial hypertrophy with a computerized imaging software (ImageJ-win32, NIH, Bethesda, MD, USA). Major and minor nuclear diameters were measured to evaluate nuclear volume (V) using the formula for a prolate ellipsoid: V = pAB2/6, where A is the major diameter and B the minor diameter [37]. Fifty nuclei from each animal were measured.

### 4.4. Immunohistochemical Evaluation of Myocardial Monocyte/Macrophage Infiltration, T Lymphocytes and Tyrosine Hydroxylase Expression

The count of monocyte/macrophage and T-lymphocytes infiltration was performed on formalin fixed and paraffin embedded transmural myocardial sections (3 µm), using respectively a monoclonal mouse anti-rat monocytes/macrophage antibody (CD68, clone ED1, MAB 1435, Chemicon, Temecula, CA, USA) as previously described [38], and a rabbit monoclonal anti-rat T-lymphocyte antibody (CD3 [SP7] ab16669, Abcam, Cambridge, UK). For each antibody, the histological sections were deparaffinized and rehydrated, treated by boiling in citrate buffer (0.01 mol/L, Ph 6) in microwave (750 W), and incubated over night at 4 °C with anti-CD68 or anti-CD3 antibody (dilution 1:300 for both antibodies). The reaction product was amplified by Ultra Tek HRP Staining System (Scy TeK Laboratories, Logan, UT, USA) and visualized with 3,3′-diaminobenzidine (DAB) (Dako, Glostrup, Denmark). Negative control was obtained by omitting the primary antibody. Sections were viewed using a Leica microscope (Leitz Camera, Wetzlar, Germany), and all slides with immunostained myocardial section for each sample were captured with Aperio scanner (Leica Biosystems, Buccinasco, MI, Italy). For each antibody, ten randomly selected images/section at ×20 magnification were analyzed. Two independent pathologists blinded to the treatment counted respectively CD68 and CD3 positive cells and took the average. The macrophages and the T-lymphocytes were expressed as mean value of positive cells/fields [38].

The tyrosine hydroxylase expression was evaluated on formalin fixed and paraffin embedded myocardial sections (3 µm) using an anti-rabbit Anti-Tyrosine Hydroxylase antibody—Neuronal Marker (ab112 Abcam, Cambridge, UK). The sections were deparaffinized and rehydrated, treated with Proteinase K (20 µg/mL; Qiagen, Hilden, Germany) for 10 min at 37 °C, and, successively incubated with primary antibody (1:1000, for an hour). The reaction product was amplified by Ultra Tek HRP Staining System (Scy TeK Laboratories, Logan, UT, USA) and visualized with 3,3′-diaminobenzidine (DAB) (Dako, Glostrup, Denmark). Negative control was obtained by omitting the primary antibody. Sections were viewed using a Leica microscope (Leitz Camera), and all slides with immunostained myocardial section for each sample were captured with Aperio scanner (Leica Biosystems). Ten randomly selected images/section at x20 magnification were analysed by two independent pathologists blinded to the treatment. The tyrosine hydroxylase immunostaining at nerve fibers was expressed as % (immunostaining area/total histological area) [38].

### 4.5. Myocardial mRNA Expression of Tyrosine Hydroxylase

Total RNA was extracted from hearts using Trizol reagent (Invitrogen, Waltham, MA, USA), following the manufacturer’s instructions. One microgram of RNA was reverse-transcribed to synthesize cDNA (High Capacity cDNA Reverse Transcription kit, Applied Biosystems, Monza, MB, Italy) and its integrity was confirmed on agarose (1%) gel stained with ethidium bromide. Myocardial mRNA expression of tyrosine hydroxylase (TH) and 18S, used as reference gene, was evaluated by real-time PCR using the QuantStudio^TM^ 7 Real Time PCR Systems (Thermofisher Scientific, Waltham, MA, USA). TH and 18S mRNA expression was evaluated using the Assay-on-Demand Gene Expression Product (Applied Biosystems), following the manufacturer’s instructions. All reactions were performed in duplicate. Expression levels were normalized to 18S mRNA expression, following the 2^−^^ΔΔ^^Ct^ formula.

### 4.6. Statistical Analysis

Data are reported as means±SEM (standard error of the mean). Differences among the groups of rats (control, Ang II, Ang II+compound 21, Ang II+Ang 1-7, Ang II+losartan) for systolic blood pressure, blood glucose, sodium, potassium, calcium, phosphate, cholesterol, triglycerides, C reactive protein, monocyte chemoattractant protein-1, body weight, heart/body weight, glomerular filtration rate, myocardial fibrosis and hypertrophy, inflammatory infiltrates, tyrosine hydroxylase expression were assessed using ANOVA followed by Fisher’s protected least-significant test for post hoc comparisons. Differences between means were considered significant at *p* < 0.05.

## Figures and Tables

**Table 1:** Systolic blood pressure (SBP, mmHg), body weight (BW, g.), heart weight (g.), heart/body weight ratio (mg/g.), glomerular filtration rate (GFR, mL/min); non fasting plasma glucose (mg/dL), sodium (mEq/L), potassium (mEq/L), calcium (mg/dL), phosphate (mg/dL), total cholesterol (mg/dL), triglycerides (mg/dL), C reactive protein (CRP, µg/mL) and monocyte chemoattractant protein (MCP-1, pg/mL) in Control (*n* = 5), Ang II (*n* = 5), Ang+C21 (*n* = 5), Ang II+Ang 1-7 (*n* = 5), Ang II+Los (*n* = 5)-treated rats at the end of the short term experimental period (1 week).

Parameters	Control	Ang II	Ang II+C21	Ang II+Ang 1-7	Ang II+Los
SBP (mmHg)	146.2 ± 2.3	198.3 ± 4.4 ‡,ζ	193.7 ± 2.3 ‡,ζ	194.3 ± 2.3 ‡,ζ	148.1 ± 1.2
BW (g)	353.5 ± 25.3	363.3 ± 2.4 δ	317.8 ± 9.7 ^	344.0 ± 2.1	372.0 ± 8.2
Heart Weight (g)	1.11 ± 0.06	1.26 ± 0.01 *^,^^	1.22 ± 0.06	1.25 ± 0.03 *	1.06 ± 0.02
Heart/Body Weight (mg/g)	3.16 ± 0.20	3.46 ± 0.05 ^	3.84 ± 0.15 †,ζ	3.63 ± 0.09 *^,^^	2.85 ± 0.05
GFR (mL/min)	1.35 ± 0.19	1.23 ± 0.02	1.30 ± 0.08	1.45 ± 0.11	1.47± 0.10
Plasma					
Glucose (mg/dL)	139.4 ± 3.5	140.8 ± 6.2	127.2 ± 6.3	129.7 ± 5.2	132.5 ± 8.5
Sodium (mEq/L)	140.0 ± 0.43	141.7 ± 0.35	139.9 ± 0.70	141.1 ± 0.80	138.2 ± 0.81
Potassium (mEq/L)	4.40 ± 0.47	4.26 ± 0.35	4.94 ± 0.66	4.20 ± 0.36	4.18 ± 0.18
Calcium (mg/dL)	7.64 ± 0.80	6.88 ± 0.18	4.75 ± 0.79	6.00 ± 0.88	7.94 ± 0.48
Phosphate (mg/dL)	6.41 ± 0.56	6.70 ± 0.59	6.93 ± 1.22	5.46 ± 0.81	6.20 ± 0.44
Cholesterol (mg/dL)	58.4 ± 4.48	65.1 ± 9.00	47.3 ± 3.3	55.3 ± 3.9	52.3 ± 4.5
Triglycerides (mg/dL)	114.2 ± 36.2	91.9 ± 21.1	63.5 ± 2.3	75.9 ± 12.7	72.6 ± 9.6
CRP (µg/mL)	34.1 ± 0.67	36.0 ± 0.63	48.2 ± 12.6	36.1 ± 5.5	43.6 ± 14.9
MCP-1 (pg/mL)	253.3 ± 6.66	287.9 ± 9.44	269.5 ± 5.85	253.4 ± 5.11	276.9 ± 23.5

Data are means ± SEM. * = *p* < 0.05 vs. Control; † = *p* < 0.01 vs. Control; ‡ = *p* < 0.0001 vs. Control; δ = *p* < 0.05 vs. Ang+C21; ^ = *p* < 0.01 vs. Ang II+Los; ζ = *p* < 0.0001 vs. Ang II+Los.

**Table 2:** Systolic blood pressure (SBP, mmHg), body weight (BW, g.), heart weight (g.), heart/body weight ratio (mg/g), glomerular filtration rate (GFR, mL/min); non fasting plasma glucose (mg/dL), sodium (mEq/L), potassium (mEq/L), calcium (mg/dL), phosphate (mg/dL), total cholesterol (mg/dL), triglycerides (mg/dL), C reactive protein (CRP, µg/mL) and monocyte chemoattractant protein (MCP-1, pg/mL) in Control (*n* = 5), Ang II (*n* = 5), Ang+C21 (*n* = 6), Ang II+Ang 1-7 (*n* = 7), Ang II+Los (*n* = 7)-treated rats at the end of the long-term experimental period (4 weeks).

Parameters	Control	Ang II	Ang II+C21	Ang II+Ang 1-7	Ang II+Los
SBP (mmHg)	145.0 ± 2.8	201.2 ± 3.9 ‡,ζ	195.4 ± 2.0 ‡,ζ	196.8 ± 4.2 ‡,ζ	138.1 ± 3.5
BW (g)	449.0 ± 11.3	369.6 ± 9.6 ‡	368.4 ± 10.9 ‡	354.0 ± 8.4 ‡	392.1 ± 13.2 †
Heart Weight (g)	1.32 ± 0.05	1.40 ± 0.02 ^	1.45 ± 0.05 ^	1.35 ± 0.03 ^	1.14 ± 0.05 *
Heart/Body Weight (mg/g)	2.94 ± 0.07	3.79 ± 0.08 †,ζ	3.94 ± 0.13 ‡,ζ	3.86 ± 0.16 ‡,ζ	2.91± 0.06
GFR (mL/min)	1.91 ± 0.19	1.41 ± 0.12 *	1.34 ± 0.14†	1.45 ± 0.12 *	1.35 ± 0.12 *
Plasma					
Glucose (mg/dL)	122.7 ± 4.8	130.0 ± 12.0	115.7 ± 7.5	113.6 ± 8.9	133.1 ± 9.6
Sodium (mEq/L)	138.6 ± 0.94	137.1 ± 2.37	138.5 ± 1.72	137.7 ± 0.91	141.2 ± 1.06
Potassium (mEq/L)	4.24 ± 0.18	3.58 ± 0.51	3.90 ± 0.33	4.62 ± 0.43	4.50 ± 0.32
Calcium (mg/dL)	7.87 ± 0.24	7.57 ± 0.46	7.65 ± 0.29	7.44 ± 0.41	7.88 ± 0.27
Phosphate (mg/dL)	6.40 ± 0.36	6.21 ± 0.65	6.18 ± 0.85	6.09 ± 0.92	7.06 ± 0.43
Cholesterol (mg/dL)	44.1 ± 2.3	49.5 ± 5.4	48.8 ± 2.3	45.3 ± 2.6	48.0 ± 2.3
Triglycerides (mg/dL)	111.9 ± 13.0	90.8 ± 20.6	91.3 ± 14.7	82.0 ± 10.3	86.7 ± 11.2
CRP (µg/mL)	31.8 ± 2.43	27.4 ± 1.85	43.9 ± 4.13	42.7 ± 8.17	43.1 ± 6.69
MCP-1 (pg/mL)	261.2 ± 7.56	270.4 ± 9.83	280.0 ± 9.36	263.5 ± 5.29	285.2 ± 19.3

Data are means ± SEM. * = *p* < 0.05 vs. Control; † = *p* < 0.01 vs. Control; ‡ = *p* < 0.0001 vs. Control; ^ = *p* < 0.01 vs. Ang II+Los; ζ = *p* < 0.0001 vs. Ang II+Los.

## Data Availability

The data presented in this study are available on request from the corresponding author.
